# Overexpression of netrin-1 increases the expression of tight junction-associated proteins, claudin-5, occludin, and ZO-1, following traumatic brain injury in rats

**DOI:** 10.3892/etm.2014.1818

**Published:** 2014-07-01

**Authors:** JIANFENG WEN, SUOKAI QIAN, QIFAN YANG, LEI DENG, YE MO, YUEFEI YU

**Affiliations:** 1 Department of Neurosurgery, Changcheng Sub-Hospital of Nanchang University, Nanchang, Jiangxi 330002, P.R. China; 2 Department of Neurosurgery, The 94^th^ Hospital of PLA, Nanchang, Jiangxi 330002, P.R. China; 3 Department of Nursing, The 94^th^ Hospital of PLA, Nanchang, Jiangxi 330002, P.R. China; 4 Department of Internal Medicine, Texas Tech University Health Sciences Center, Lubbock, TX 79430, USA

**Keywords:** traumatic brain injury, blood-brain barrier, tight junctions, netrin-1

## Abstract

The function of the blood-brain barrier (BBB) depends on the integrity of tight junction (TJ)-associated proteins. Netrin-1 is known to promote angiogenesis and may also regulate the BBB. To understand the association between netrin-1 and the TJ-associated proteins, the expression levels of proteins involved in maintaining the integrity of the BBB, including netrin-1, claudin-5, occludin and zonula occluden (ZO)-1, were investigated in the present study using quantitative polymerase chain reaction, western blot analysis and immunofluorescence. The aim of the present study was to determine the changes in BBB permeability and whether pZsGreen1-N1 mediated overexpression of netrin-1 increased the expression of the TJ-associated proteins following traumatic brain injury (TBI). The results demonstrated that the levels of mRNA transcription and protein expression of the TJ-associated proteins, claudin-5, occludin and ZO-1, were significantly reduced following TBI. Furthermore, the changes in the expression of these three TJ proteins were consistent with the changes in the BBB permeability, indicating that weakening intercellular junctions leads to BBB opening. The present study also demonstrated that netrin-1 significantly increased the downregulation of claudin-5, occludin and ZO-1 expression levels induced by TBI, which provided a basis for further investigation on the role of netrin-1 in the integrity of TJs and proper functioning of the BBB.

## Introduction

Traumatic brain injury (TBI) is the main cause of mortality and disability in young individuals, and can directly cause pathophysiological changes in the blood-brain barrier (BBB). The BBB is primarily comprised of brain microvascular endothelial cells, the basement membrane and glial cells surrounding the capillaries. The endothelial cells come into contact with each other at what are known as tight junctions (TJs). TJs consist of the transmembrane proteins, occludins and claudins, that interact on adjacent endothelial cells to form a physical barrier against paracellular diffusion (1–3), and the accessory proteins, zonula occludens (ZO) family (ZO-1 and ZO-2), that anchor the transmembrane proteins to the cytoskeleton (4–6).

Netrin-1, one of three members in the mammalian netrin family, stimulates angiogenesis and augments the response to vascular endothelial growth factor (7). In addition, netrin-1 has been found to be superior to vascular endothelial growth factor in restoring nerve conduction velocity, possibly due to the potent effects on vascular and neural biology (8). Notably, netrin-1 may also play a role in the restoration of the BBB. The angiogenic effect of netrin-1 offers unique therapeutic potentials in restoring the BBB under pathological conditions, including TBI.

Although the disruption of the BBB has been previously investigated in several TBI models (9,10), there is limited information with regard to the association between netrin-1 and TJs. Therefore, the aim of the present study was to analyze the correlation between netrin-1 and TJs in a TBI model.

## Materials and methods

### Animal models

In total, 20 male Sprague-Dawley rats (weight, 250–280 g) were used in the study (10 rats in the TBI group and 10 rats in the sham-operated group). All animal procedures were approved by the Nanchang University Medical School Animal Care and Use Committee (Nanchang, China). The experimental TBI model was established as previously described by Feeney *et al* (11). Briefly, the animals were anesthetized via intramuscular injection of xylazine/ketamine HCl (10/90 mg/kg). The head was then fixed in a stereotactic frame along the midline incision scalp, and periosteal stripping was performed to expose the left parietal region. A bone window measuring 5 mm in diameter was established at 1.5 cm posterior to the bregma and 2.5 mm beside the midline. A 40-g metal sterile rod fell freely from a height of 30 cm to hit the duramater and create a contusion in the left parietal lobe. The bone window was then closed with bone wax and the scalp incision sutured, following which the animals were removed from the stereotactic frame. The body temperature was maintained at 37±0.5°C using a heating pad during the surgical procedure. The animals were sacrificed at 72 h following TBI and the ipsilateral cortices were removed intact. Tissues were dissected immediately and stored in liquid nitrogen until required for further analysis.

### Measurement of Evans blue (EB) dye extravasation

BBB permeability was quantitatively evaluated using the extravasation of EB dye as a marker of albumin extravasation (12). Briefly, 2% EB dye was slowly injected intravenously 2 h prior to sacrifice. At 24, 48, 72 and 96 h following TBI, the rats were deeply anesthetized with 10% chloral hydrate and perfused with heparinized saline via the cardiac ventricle until colorless perfusion fluid was obtained from the atrium. The ipsilateral cortex was quickly removed and placed on ice, and a coronal section of the injured hemisphere through the impact site was dissected using a double-blade scalpel. Brain samples were weighed and then immersed in 5 l/kg formamide at 50°C for 72 h. The supernatant was collected and the fluorescence was measured using a multiplate reader (Synergy; BioTek, Inc., Winooski, VT, USA). The fluorescent intensity was normalized against wet tissue weight, and the EB dye tissue content was quantified based on a linear standard line.

### Immunofluorescence

Ipsilateral cortices were removed and post-fixed overnight in 4% paraformaldehyde at 4°C. Immunofluorescence signals of occludin, claudin-5 and ZO-1 were then determined in perfused-fixed paraffin-embedded sections. The paraffin-embedded sections were deparaffinized and placed through a series of alcohols with decreasing concentrations. Slides were blocked for 1 h at room temperature and incubated in anti-claudin-5, anti-occludin and anti-ZO-1 antibodies (1:200; Santa Cruz Biotechnology, Inc., Dallas, TX, USA) at 4°C overnight. The slides were then rinsed three times for 5 min in phosphate-buffered saline containing 0.1% Tween-20, and incubated with secondary anti-rabbit immunoglobulin G, conjugated with Alexa Fluor 488 (Invitrogen Life Technologies, Carlsbad, CA, USA), for 30 min. For image analysis, the slides were mounted following subsequent washing procedures and examined under an Olympus BX-51 epifluorescence microscope (magnification, ×100; Olympus, Tokyo, Japan).

### Western blot analysis

Cell suspensions were prepared from dissected ipsilateral cortices and transferred to a fresh tube. The suspensions were homogenized using a handheld mortar and pestle and then agitated for 10 min. Extracts were clarified by centrifugation and then diluted in a reducing agent. Proteins were resolved on a 12% Bis-Tris polyacrylamide gel and electrotransferred onto a nitrocellulose membrane. The membrane was incubated with anti-claudin-5, anti-occludin or anti-ZO-1 antibodies (1:200; Santa Cruz Biotechnology, Inc.), followed by incubation with goat-anti-rabbit horseradish peroxidase-conjugated secondary antibodies (1:1,000; Santa Cruz Biotechnology, Inc.) in blocking buffer. The membrane was then developed using enhanced chemiluminescence detection and film exposure (Amersham, Little Chalfont, UK). GAPDH was used as the internal control.

### Quantitative polymerase chain reaction (qPCR)

Total RNA was isolated from the tissues of ipsilateral cortices using TRIzol reagent (Gibco-BRL, Gaithersburg, MD, USA), and treated with 200 units Moloney Murine Leukemia Virus reverse transcriptase (Promega Corporation, Madison, WI, USA) for first-strand cDNA synthesis. A SYBR Green Detection kit (Ameritech Biomedicines, Houston, TX, USA) and Applied Biosystems Prism 7500 detection system (Applied Biosystems, Foster City, CA, USA) were used to amplify the transcribed cDNA for 40 cycles. The real time thermal cycler program consisted of three stages: Stage one, 95°C for 5 min; stage two, 94°C for 20 sec, followed by 57°C for 20 sec and 72°C for 20 sec (repeated 40 times); and stage three, 72°C for 5 min, followed by 55°C for 10 sec and 95°C for 15 sec. The copy numbers of netrin-1, claudin-5, occludin and ZO-1 mRNA were normalized against the internal control, β-actin. The sequences of the PCR primers used were as follows: Netrin-1 forward, 5′-CTACTGCAAGGAGGGCTTCTA-3′ and reverse, 5′-GCGCTACAGGAATCTTAATG-3′; occludin forward, 5′-ACAAAGAGCTCTCTCGTCTCG-3′ and reverse, 5′-CATAGTCTCCCACCATCCTC-3′; claudin-5 forward, 5′-CACAGAGAGGGGTCGTTGAT-3′ and reverse, 5′-CTGCCCTTTCAGGTTAGCAG-3′; ZO-1 forward, 5′-AGTTCTGCCCTCAGCTACCA-3′ and reverse, 5′-GCTTAAAGCTGGCAGTGTC-3′; and β-actin forward, 5-CCTAGACTTCGAGCAAGAGA-3′ and reverse 5′-AGAGGTCTTTACGGATGTCA-3′.

### Microvessel isolation

Ipsilateral cortices from rats in the TBI group were removed and homogenized, prior to being transferred to a 40-ml syringe with a 300-μm nylon mesh. The homogenates were filtered through the mesh, which was repeated with a 115-μm nylon mesh. The filtrate was transferred to a graduated cylinder with an equal volume of 40% dextran, and centrifuged for 15 min at 5,000 × g. The supernatant was carefully aspirated, following which the pellet was resuspended and filtered through a 20-μm nylon mesh. The filter was inverted and rinsed in a Petri dish to remove the microvessels. Finally, the brain microvascular endothelial cells were cultured in rat brain endothelial cell growth medium (Cell Applications, Inc., San Diego, CA, USA).

### Construction of the netrin-1 gene delivery system

A pZsGreen1-N1-netrin-1 vector (with netrin-1 gene insert) was prepared as previously described (13). A pZsGreen1-N1 vector without the netrin-1 insertion was used as a control vector. Microvascular endothelial cells from the ipsilateral cortex were transfected with the recombinant pZsGreen1-N1-netrin-1 plasmid or the empty vector using Lipofectamine 2000 (Invitrogen Life Technologies, Grand Island, NY, USA).

### Statistical analysis

Data are expressed as the mean ± standard error of the mean. The results were analyzed with GraphPad Prism version 4.00 for Windows software (GraphPad Software, Inc., San Diego, CA, USA), using the Student’s t-test and one-way analysis of variance. P<0.05 was considered to indicate a statistically significant difference. Post-hoc pairwise comparisons were performed using Tukey’s method.

## Results

### TBI markedly increases extravasation of EB dye

**Figure 1:**
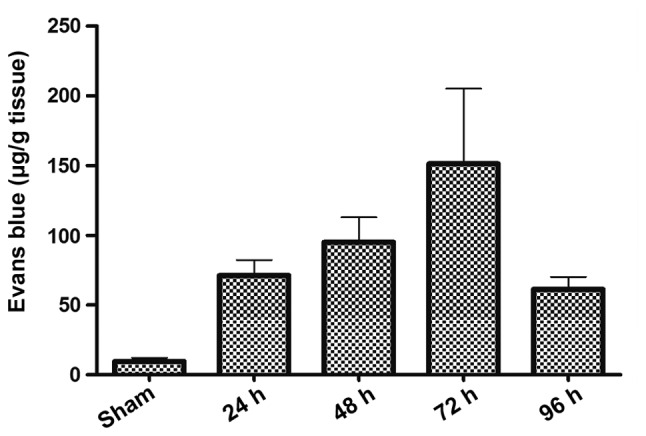
Measurement of extravasated EB dye. EB dye accumulation was significantly increased in the TBI group (n=20), but not in the sham-operated rates (n=5). Data are expressed as the mean ± standard error of the mean. EB, Evans blue; TBI, traumatic brain injury.

EB dye does not permeate an intact BBB; however, following TBI, EB dye easily permeates a compromised BBB. The severity of BBB disruption at the site of TBI, expressed as EB dye extravasation per gram of hemispheric tissue, is shown in Fig. 1. EB dye extravasation was significantly increased following TBI when compared with the sham-operated group. A peak increase in permeability was observed at 72 h following TBI.

### TBI reduces the expression of TJ proteins

**Figure 2:**
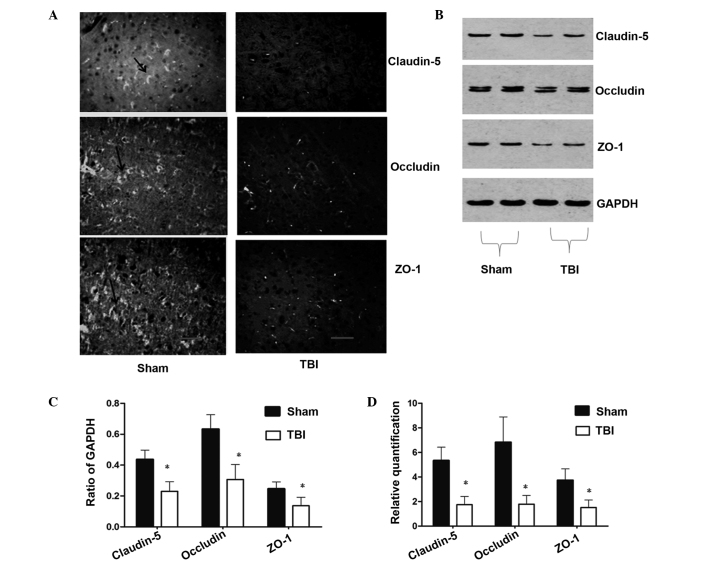
Changes in the expression levels of claudin-5, occludin and ZO-1 at 72 h following TBI. (A) Distribution and expression of claudin-5, occludin and ZO-1 in the ipsilateral cortex, as shown by immunofluorescence (magnification, ×100; scale bar, 50 μm). (B) Western blot analysis revealed reduced expression levels of claudin-5, occludin and ZO-1 in the ipsilateral cortex following TBI. (C) Densitometric analysis of the results from the western blot analysis, where the data were normalized against GAPDH expression. (D) qPCR analysis demonstrated that the expression levels of claudin-5, occludin and ZO-1 in the ipsilateral cortex were significantly reduced at 72 h following TBI when compared with the sham-operated rats. Data are expressed as the mean ± the standard error of the mean. ^*^P<0.05, vs. sham-operated groups. ZO, zonula occluden; TBI, traumatic brain injury; qPCR, quantitative polymerase chain reaction.

To investigate the effect of TBI on TJ proteins, immunofluorescence and western blot analysis were performed to detect the protein expression levels. Immunofluorescence analysis of TJ-associated proteins demonstrated changes in the localization and expression levels of claudin-5, occludin and ZO-1 following TBI in the rats. As shown in Fig. 2A, marked staining for claudin-5, occludin and ZO-1 (as shown by the black arrow) was observed at the cell-cell junctions in the sham-operated group, while fluorescent staining of the interendothelial TJ-associated proteins was reduced in the TBI group at 72 h. As shown in Fig. 2B and C, the results from the western blot analysis were consistent with the observations obtained from the immunofluorescence analysis. The expression levels of claudin-5, occludin and ZO-1 in the TBI group were significantly lower compared with those in sham-operated group. These results demonstrated that TBI reduced the expression of TJ-associated proteins.

The qPCR results demonstrated that the mRNA expression levels of claudin-5, occludin and ZO-1 were significantly reduced in the TBI group when compared with the sham-operated group (P<0.05). Compared with sham-operated group, the relative gene expression levels of claudin-5, occludin and ZO-1 were decreased by 67.17, 73.76 and 59.25%, respectively (Fig. 2D), in the TBI group.

### pZsGreen1-N1-netrin-1 gene transfer increases the expression of claudin-5, occludin and ZO-1 following TBI

**Figure 3:**
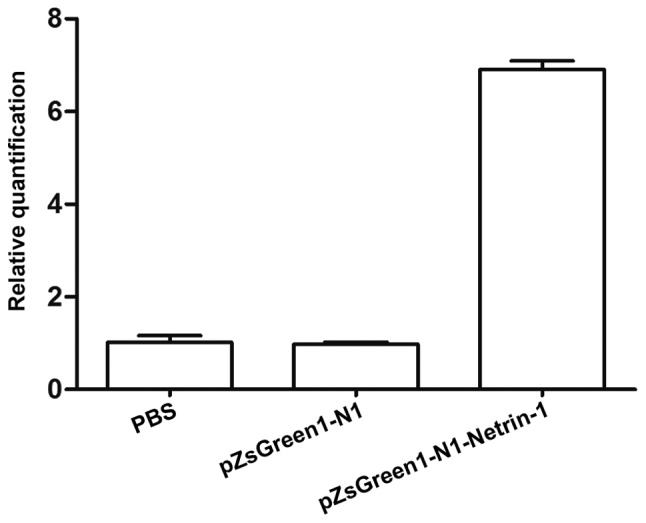
Netrin-1 mRNA expression levels increased in endothelial cells following pZsGreen1-N1-netrin-1 transfection. β-actin was used as the internal control. Data are expressed as the mean ± standard error of the mean. P<0.05, pZsGreen1-N1-netrin 1 vs. controls (PBS and pZsGreen1-N1). PBS, phosphate-buffered saline.

**Figure 4:**
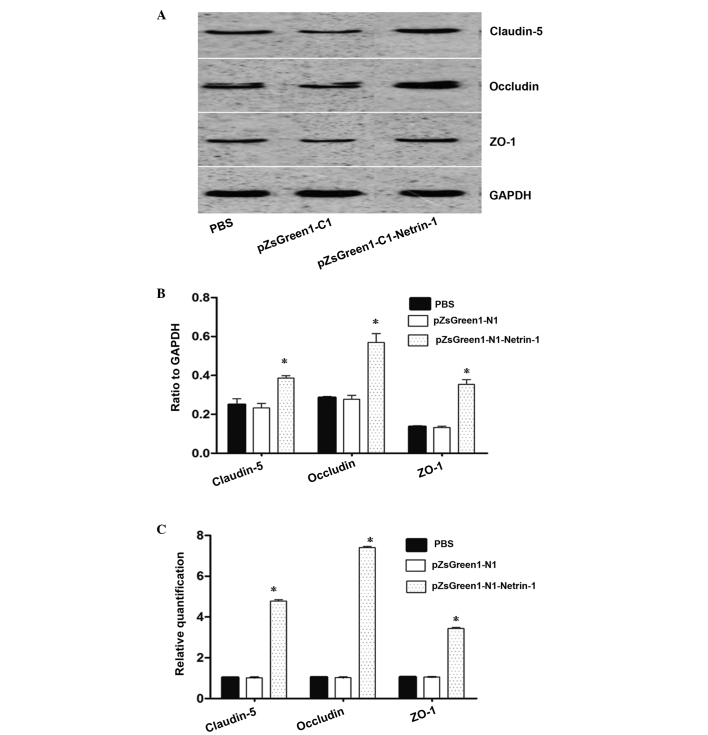
Expression levels of claudin-5, occludin and ZO-1 were higher in pZsGreen1-N1-netrin-1-transfected endothelial cells. (A) Protein expression levels of claudin-5, occludin and ZO-1, as determined by western blot analysis. (B) Densitometric analysis of the results from the western blot analysis showing the increased expression levels of claudin-5, occludin and ZO-1 in the brain endothelial cells from rats in the TBI group following pZsGreen1-N1-netrin-1 transfection. (C) mRNA expression levels of claudin-5, occludin and ZO-1 in the netrin-1 gene transfected endothelial cells of rats in the TBI group were significantly higher compared with the control group. β-actin was used as the internal control. Data are expressed as the mean ± standard error of the mean. ^*^P<0.05, vs. controls (PBS and pZsGreen1-N1). PBS, phosphate-buffered saline; ZO, zonula occluden; TBI, traumatic brain injury.

As shown in Fig. 3, the mRNA expression levels of netrin-1 in the endothelial cells transfected with pZsGreen1-N1-netrin-1 were significantly higher compared with the control group. To assess the association between netrin-1 and claudin-5, occludin and ZO-1 expression, the expression levels of TJ proteins in endothelial cells from TBI rats transfected or non-transfected with pZsGreen1-N1-netrin-1 were analyzed. The results from Fig. 4A and B indicate that the protein expression levels of claudin-5, occludin and ZO-1 in the brain microvascular endothelial cells increased following pZsGreen1-N1-netrin-1 transfection. Compared with the non-transfected and control-vector-treated endothelial cells, there was a >3–6 fold increase in the mRNA expression levels of claudin-5, occludin and ZO-1 in the pZsGreen1-N1-netrin-1 treated cells (Fig. 4C).

## Discussion

The ability to maintain the BBB integrity depends on adequate structural support from the TJ-associated proteins, which include claudin-5, occludin and ZO-1. Occludin was the first integral membrane protein identified within the TJs of endothelial cells. A deletion construct lacking the N terminus and extracellular domains of occludin has been shown to exhibit a marked effect on TJ integrity (14). Occludin has an important role in maintaining TJ assembly and barrier function. Transmembrane protein, claudin-5, has also been shown to directly regulate the integrity and proper functioning of the BBB (15,16). For example, mice with a claudin-5 deletion succumb as neonates due to the size-selective loosening of the BBB for molecules <800 Da (17). Drugs that increase claudin-5 expression increase transendothelial electrical resistance and decrease the BBB permeability (18). ZO-1 is a peripheral protein localized at junction sites that interacts directly with the majority of TJ transmembrane proteins, including occludins and claudins. Epithelial cells deficient in ZO-1 do not form TJs due to the lack of claudin polymerization (19), and delayed barrier establishment (20). Therefore, ZO-1 appears to be crucial for the formation and function of TJs. TJ-associated proteins, including claudin-5, occludin and ZO-1, have critical roles in the maintenance of BBB functions; thus, the expression levels of these TJ proteins following TBI were investigated in the present study. The results demonstrated that the levels of mRNA transcription and protein expression of these three TJ-associated proteins were significantly reduced following TBI (Fig. 2). Furthermore, BBB permeability was markedly increased in the injured brain regions following TBI when compared with the sham-operated group, as shown by the results from the EB dye extravasation (Fig. 1). These observations indicate that the changes in the distribution and the decreased expression levels of claudin-5, occludin and ZO-1 were consistent with the results of the BBB permeability changes following TBI.

Netrin-1 hyperstimulation is able to promote focal neovascularization in the adult brain *in vivo* (21). In addition, Liu *et al* (22) previously demonstrated that netrin-1 may regulate the BBB (22). Thus, the various roles of netrin-1 may engage in the recovery processes of TBI. Understanding the mechanisms underlying TJ-associated proteins development and functioning, and more specifically the effects that netrin-1 exhibits on the BBB, is of utmost importance. In the present study, the association between netrin-1 and the expression levels of TJ-associated proteins was investigated. The results revealed that pZsGreen1-N1-mediated netrin-1 transcription was detected in brain microvascular endothelial cells at an mRNA level following gene transfer. Notably, a significant enhancement in the expression levels of claudin-5, occludin and ZO-1 were observed in the endothelial cells isolated from TBI following pZsGreen1-N1-netrin-1 gene transfer (Fig. 4). Since brain endothelial cells play a critical role in the structural and transport maintenance of the BBB, and the BBB permeability depends on the integrity of the TJs and the expression of claudin-5, occludin and ZO-1, the results from the present study indicate that overexpression of netrin-1 may improve the TJs of brain endothelial cells and contribute to the recovery of the BBB following TBI. The development of novel therapies for the treatment of TBI may involve netrin-1 in the repair of the BBB; thus, future study should focus on the association between netrin-1 and the integrity of TJs and the recovery of the BBB.

In conclusion, overexpression of netrin-1 increases the expression levels of TJ-associated proteins following TBI, which provides a solid foundation for further study investigating the role of netrin-1 in the integrity of TJs and the function of the BBB.
